# A high-resolution structure of the EF-hand domain of human polycystin-2

**DOI:** 10.1002/pro.2513

**Published:** 2014-07-02

**Authors:** Mark D Allen, Seema Qamar, Murali K Vadivelu, Richard N Sandford, Mark Bycroft

**Affiliations:** 1 MRC Laboratory of Molecular BiologyHills Road, Cambridge, CB2 0QH, United Kingdom; 2 Department of Clinical Neurosciences, Cambridge Institute for Medical Research, University of CambridgeCambridge, CB2 0XY, United Kingdom; 3 Academic Department of Medical Genetics, School of Clinical Medicine, University of CambridgeCambridge, CB2 0QQ, United Kingdom

**Keywords:** ADPKD, polycystin-2, EF-hand, solution structure, NMR, ITC, mutagenesis

## Abstract

Autosomal dominant polycystic kidney disease (ADPKD) affects over 1:1000 of the worldwide population and is caused by mutations in two genes, *PKD1* and *PKD2*. *PKD2* encodes a 968-amino acid membrane spanning protein, Polycystin-2 (PC-2), which is a member of the TRP ion channel family. The C-terminal cytoplasmic tail contains an EF-hand motif followed by a short coiled-coil domain. We have determined the structure of the EF-hand region of PC-2 using NMR spectroscopy. The use of different boundaries, compared with those used in previous studies, have enabled us to determine a high resolution structure and show that the EF hand motif forms a standard calcium-binding pocket. The affinity of this pocket for calcium has been measured and mutants that both decrease and increase its affinity for the metal ion have been created.

## Introduction

Autosomal dominant polycystic kidney disease (ADPKD) is a common inherited renal disease, with a worldwide prevalence of 1:1000 and is frequently complicated by cardiovascular disease including hypertension and cerebral aneurysm. It typically causes end-stage renal failure (ESRF) in the 6th to 8th decades of life requiring renal replacement therapy such as dialysis or transplantation.^1^ About 85% of cases of ADPKD are caused by mutations in *PKD1* with the remainder due to mutations in *PKD2*.^2^ *PKD1* encodes polycystin-1 (PC-1), a large multi-membrane spanning glycoprotein of 4302 residues and mass >460 kDa. It has a short 198 amino acid intracellular C-terminus that contains a coiled-coil domain that interacts with the C-terminus of polycystin-2 (PC-2), the protein product of *PKD2* gene.^3^ Mutations of *PKD2* that result in the loss of functional polycystin-2 in renal tubular and biliary epithelial cells cause these cells to proliferate, lose their normal structure within tissue architecture, and go on to form cysts.

PC-2 is the prototypic member of the TRP (transient receptor potential) channel family,^4^,^5^ and is expressed in most tissues. The PC-1:PC-2 complex functions as a mechanosensitive Ca^2+^ ion channel that localizes to primary cilia.^6^ PC-2 consists of six transmembrane helices, with both the N- and C-termini being cytoplasmic. The C-terminal cytoplasmic portion of PC-2 consists of three regions: an EF-hand motif (711–787), a flexible linker 787–827, and a coiled-coil domain that mediates the interaction with PC-1 (828–895).^7^ EF hands motifs can bind calcium and often mediate responses to changes in calcium concentration. The EF hand motif in PC-2 has been shown to play an important role in modulating channel gating. This region of PC-2 has previously been investigated using NMR spectroscopy but these studies have given conflicting results.^8^,^9^ As part of a long-standing study of the structure and function of the polycystins,^10^ we have determined a high resolution NMR structure of the PC-2 EF-hand domain. This has allowed us to determine how the domain binds calcium and make a detailed comparison to the structures of other EF-hand domains.

## Results

### Structural studies of the C-terminal EF-hand domain of PC-2

**Figure 1:**
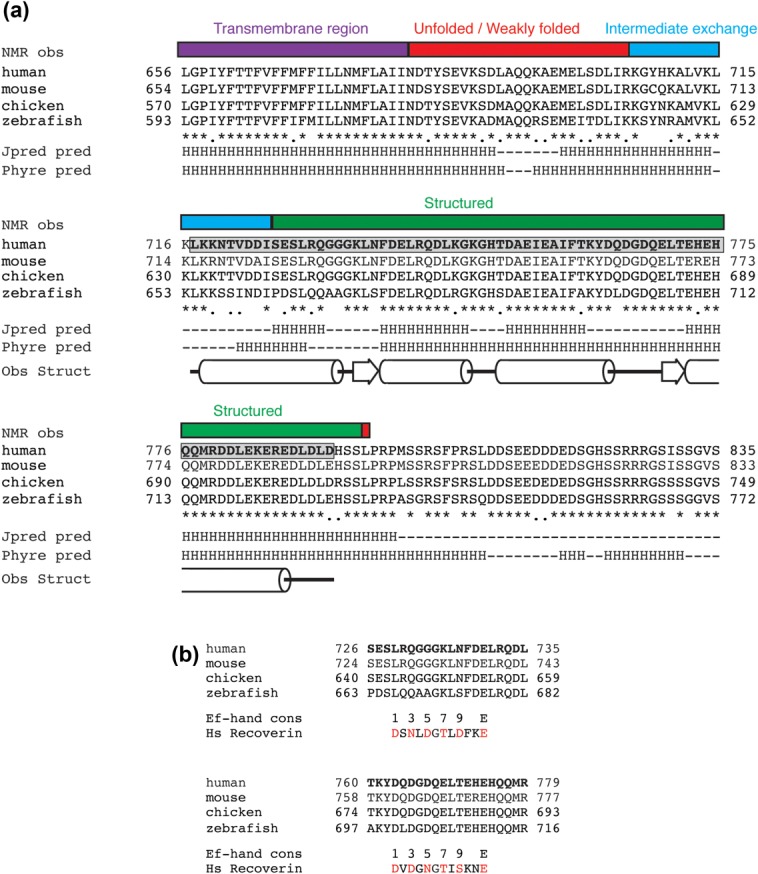
(a) An alignment of the polycystin-2 sequences from vertebrates. A representation of the NMR behavior observed by Schumann *et al*. and the predicted transmembrane region is illustrated above the alignment. Conservation of residues is indicated for identity (*) and conserved (.) residues. The sequence of the domain used for structure determination is shaded in grey. The secondary structure predictions of both Jpred and Phyre are indicated below the alignment. A diagrammatical representation of the determined structure is also shown. (b) An alignment of the polycystin-2 putative EF-hand motifs from vertebrates. The residues important for calcium binding are indicated, along with their positions in the EF-hand motif. The sequences of the EF-hand motifs of human recoverin are shown and the residues involved in calcium binding are colored red.

A series of fragments of the C-terminal cytoplasmic region of PC-2 were cloned, expressed and their suitability for NMR studies determined. Constructs containing the C-terminal coiled-coil were found to precipitate upon the addition of calcium and were not investigated further. Focusing on the region around the EF hand motif, we found that sequence alignment and secondary structure prediction [Fig. 1(a)] suggested that it was embedded with a larger structured region (residues 680–792). NMR spectra were recorded of a series of constructs encompassing this region in absence and presence of 5 m*M* calcium and the peptide corresponding to residues 717–792 was found to be most suitable for NMR studies. Additional residues at the N-terminus caused the domain to be less stable and hence these constructs were not used. These findings were in agreement with those of Schumann *et al*. who used a clone from 680 to 796 and observed the region from 680 to 705 to be characterized by somewhat broad lines, chemical shifts close to those observed for random coil amino acids and NMR relaxation data consistent with a weakly structured region.^8^ Furthermore, Schumann *et al*. were unable to detect resonance for residues from 707 to 724, suggesting this region to be in intermediate exchange. It is possible that the region from 680 to 717 can become structured upon interacting with either the membrane or a functionally important protein, but under the conditions used the region was not sufficiently ordered to obtain structural information. Interestingly, two structures of ion-channels identified by PHYRE to have homology to polycystin-2 were shown to have helical motifs in this region,^11^,^12^ suggesting that the ion channel may be linked to the EF-hand motif by a helical extension.

**Figure 2:**
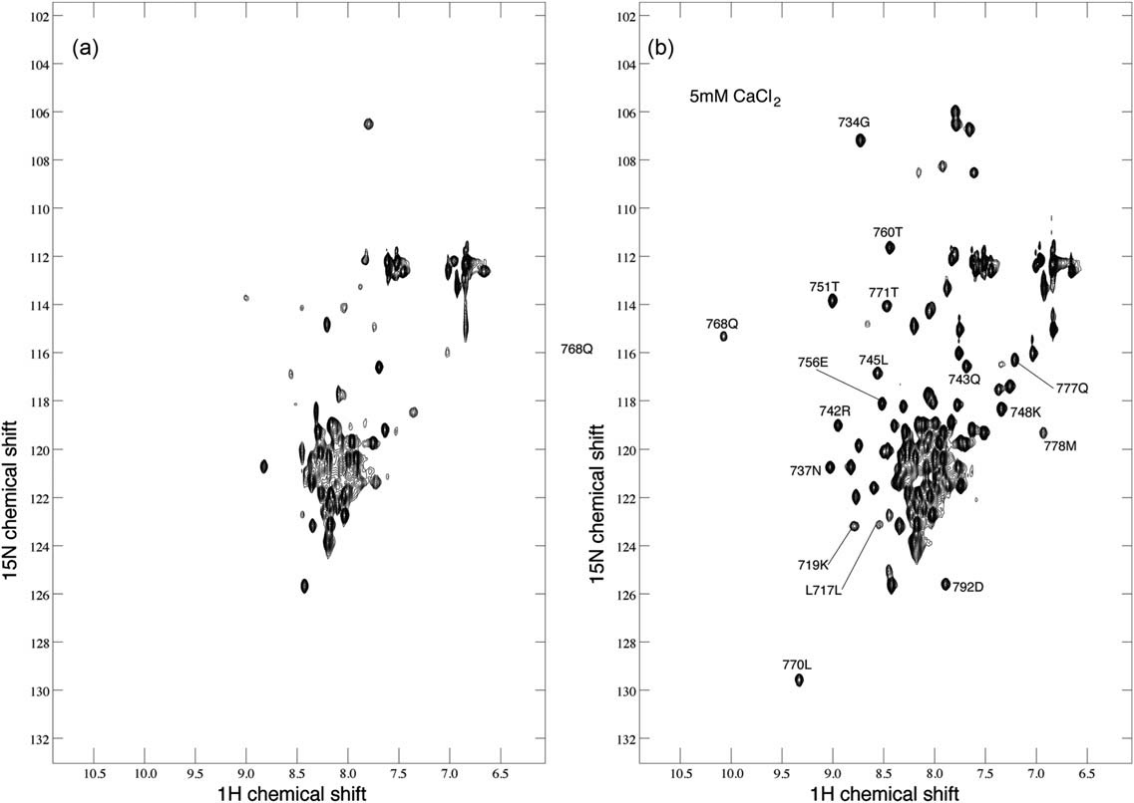
^1^H-^15^N HSQC spectra of human polycstin-2 in the absence of calcium (a) and the presence of 5 m*M* calcium (b). Some of the down-fielded shift residues are indicated.

^15^N-^1^H HSQC measurements of PC-2 EF-hand indicated that this region of the protein has the characteristics of a molten-globule in the absence of calcium [Fig. 2(a)], but upon addition of calcium the spectrum shows dispersion typical of a folded domain [Fig. 2(b)]. Using ITC it was found that PC-2 717-792 binds a single calcium ion with an affinity of 122 ± 5.2 µ*M*. Calcium binding was abolished when glutamate 774 within the EF hand motif was mutated to glutamine. Our domain boundaries appear to have resolved the protein aggregation and NMR relaxation problems noted elsewhere for this domain and allowed a complete ^1^H assignment of the domain and the identification of a large number of long range NOEs.

An initial model of the domain was calculated by CS-Rosetta (Supporting Information Fig. S2) from the chemical shift and RDC measurements. The structures calculated with and without RDCs were essentially the same with the lowest energy structures having an r.m.s.d. of 1.01 Å over residues 717–785. CNS was subsequently used to determine a high-resolution solution structure of the domain using NOE, dihedral angle, hydrogen-bond and residual dipolar coupling (RDC) constraints. Structures were initially determined with a calcium ion not included in the structure calculations to prevent bias. Once an unambiguous calcium-binding pocket was seen (Supporting Information Fig. S3), a calcium ion was incorporated into structure calculation based on the coordination observed in other EF hands using established protocols. A summary of all conformational constraints and statistics is presented in Table I.

**Table I:** Summary of Conformational Constraints and Statistics for the 20 Accepted NMR Structures of PC-2 EF-Hand Domain

Structural constraints	
Intra-residue	660
Sequential	316
Medium-range (2 ≤ |i–j| ≤ 4)	262
Long-range (|i–j| > 4)	326
Dihedral angle constraints	18
TALOS constraints	134
RDC constraints	50
Distance constraints for 19 hydrogen bonds	38
Calcium co-ordination constraints	6
Total	1810
Statistics for accepted structures	
Statistical parameters (±SD)	
Rms deviation for distance constraints	0.0072 ± 0.0010 Å
Rms deviation for dihedral constraints	0.200 ± 0.016°
Mean CNS energy term (kcal mol^−1^ ±SD)	
E (overall)	93.02 ± 9.16
E (van der Waals)	21.33 ± 3.48
E (distance constraints)	6.30 ± 2.09
E (dihedral and TALOS constraints)	1.48 ± 0.24
E (RDC constraints)	14.77 ± 1.69
Rms deviations from the ideal geometry (±SD)	
Bond lengths	0.0014 ± 0.0001 Å
Bond angles	0.341 ± 0.0105°
Improper angles	0.278 ± 0.013°
Average atomic rmsd from the mean structure (±SD)	
Residues 717–785 (N, Cα, C atoms)	0.264 ± 0.064 Å
Residues 717–785 (all heavy atoms)	0.864 ± 0.050 Å

**Table II:** Affinity of the WT PC-2 EF-Hand and Mutant PC-2 EF-Hand to Divalent Cations

Protein	Kd µM (±SD) [Cation]
WT EF	122 ± 6.1 [Ca^2+^]
WT EF	90 ± 5.3 [Mg^2+^]
E740Q	139 + 6.2 [Ca^2+^]
E774Q	No binding [Ca^2+^]
E740Q/E774Q	No binding [Ca^2+^]
Q768G	12 + 0.2 [Ca^2+^]

A comparison of our structure with those determined previously (Fig. 4) reveals similarity to that determined by Petri^9^ and colleagues with an r.m.s.d. of 4.0 Å for the backbone residues (720–785) of 2KQ6. The construct used by Petri was shorter at the N-terminus than the one used here probably as a result of the use of limited proteolysis to determine the domain boundaries. We observe several long range NOEs from residues missing in their construct, in particular from L717. The removal of these residues may have also affected the stability of their protein, and consequently the quality of the spectra, resulting in a low number of long-range NOEs in their calculations (41 for the 78 residues construct). Superimposition of the domain using residues 734–785 reveals a greater degree of agreement with an r.m.s.d. of 2.21 Å. There are, however, significant differences in the position of the side-chains of residues known to be important for calcium binding [Fig. 4(d,f)]. The large number of NOEs determined for our structure enabled the side-chains in the EF-hand to be constrained in positions consistent with calcium binding (Supporting Information Fig. 3).

**Figure 3:**
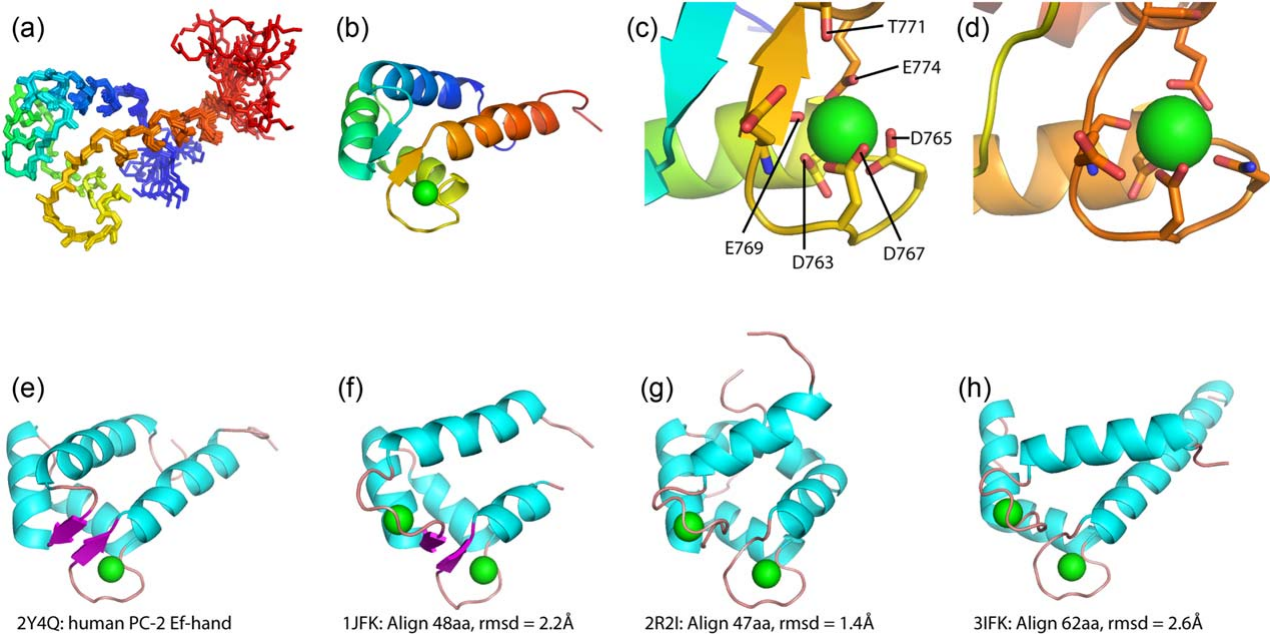
(a) Superimposition of the final 20 energy-minimized conformers of human PC-2 EF-hand domain (2Y4Q). (b) A ribbon representation of human PC-2 EF-hand domain. The position of the calcium ion is shown. (c) A detailed representation of the EF-hand motif from human PC-2. The residues involved in calcium binding are shown in relation to the calcium ion incorporated in the structure calculation. (d) A detailed representation of the EF-hand motif from chicken guanylyl cyclase-activating protein 1 (2R2I) in the same orientation. (e-h) Secondary structure representations of structural homology hits determined by VAST to the human PC-2 EF-hand domain (e): a calcium-binding protein from *amoeba histolytica* (f), guanylyl cyclase-activating protein 1 from chicken (g) and a N-terminal fragment of calmodulin from rat (h). The PDB codes, length of alignment and r.m.s.d. to human PC-2 are indicated.

**Figure 4:**
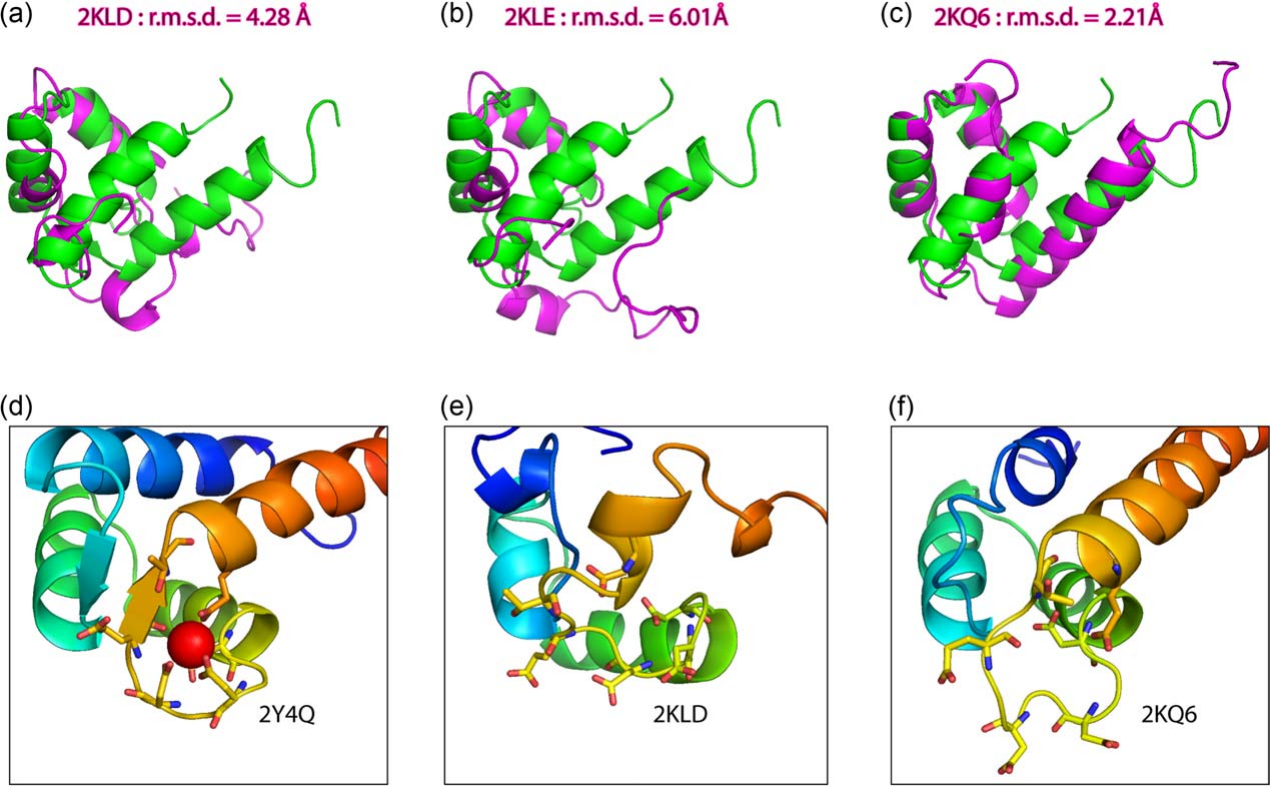
Superimposition of human PC-2 EF-hand domains. Ribbon representation of 2Y4Q superimposed with the structures of (a) 2KLD, (b) 2KLE, and (c) 2KQ6. 2KLD and 2KLE were superimposed over residues 724–785, whilst 2KQ6 was superimposed over residues 734–785. Ribbon representation of EF-hand motif of human polycystin-2 EF-hand domain 2Y4Q (d), 2KLD (e), and 2KQ6 (f). Residues involved in binding calcium are shown. The position of the calcium ion is shown for 2Y4Q.

Our structure shows less similarity to those determined by Schumnann *et al*.^8^ with an r.m.s.d. of 4.28 and 6.02 Å for 2KLD and 2KLE, respectively. The 2KLD structures were determined using CNS whilst 2KLE was determined using a protocol involving AUREMOL-ISIC which uses known structures to assist in refinement. The construct used for the 2KLD and 2KLE structures were considerably longer than those of Petri (680–796) but unfortunately the spectra did not allow for the assignments for residues 707–724. As such, the N-terminus of the structures determined by Schumann *et al*. is undefined and this possibly contributed to the lack of definition at the C-terminus due to the loss of long-range NOEs between these regions. Whilst there are more NOEs used in the calculation of 2KLD and 2KLE structures, the EF-hand region of the protein is also somewhat undefined [Fig. 4(e)] with several of the side-chains adopting conformations inconsistent with calcium binding. Our structure provides the first detailed information on the structure of the EF-hand motif of the PC-2 domain and thus provides a framework for further analysis of the mechanism of calcium sensing.

Proteins usually contain at least two EF hand motifs, typically with two motifs interacting to form a compact αβααβα fold in which the calcium ions are bound by residues in the loops between the first and second and the third and four helices. The PC-2 domain has a similar fold although in this case only the loop between the third and fourth helices binds a calcium ion. The loop between the first two helices that would normally bind a second calcium ion is truncated in this domain. Although it still forms a short stretch of sheet with residues in the canonical EF hand loop, a key feature of EF hand proteins, several of the residues that would normally bind a calcium ion are missing [Fig. 1(b)]. A glutamate residue is present at the start of helix 3 in what would normally be the final position of an EF hand motif but mutation of this residue to glutamine has no effect on calcium binding.

The calcium affinity of the PC-2 EF hand domain is rather low for this class of protein (Table II). We noted that residue Q768, at position 6 in the canonical EF-hand motif, adopts an unusual phi angle not typically associated with nonglycine residues. Given that position 6 of an EF-hand is most often a glycine we made a Q768G mutant to observe the effect on calcium binding. Strikingly the Q768G mutant was able to bind calcium 10-fold tighter than the wild-type EF-hand domain (12 ± 0.2 µ*M* and 122 ± 6.1 µ*M*, respectively). This is possibly a result of the linkage in this domain between folding and binding as it is more energetically favorable for glycine to adopt this backbone conformation.

Members of the EF-hand superfamily are typically divided into three conformations: open, closed, and semi-open, depending on the relative orientation of the helices.^13^ Calcium–free EF-hand motifs are invariably found in the “closed” conformation. Upon binding calcium, a rearrangement of the helices in the motif can occur that leads to an “open” conformation in which a large hydrophobic surface is exposed that has the potential to bind to target proteins or ligands. The “semi-open” conformation has to date only been found in myosins.^14^ Calcium-induced conformation changes are common in EF-hand proteins, including calmodulin, that have a role in signal transduction proteins. Not all EF-hand containing proteins; however, undergo large conformational change upon binding calcium and these are thought to simply buffer calcium concentration.^15^

The final three helices of the PC-2 domain are very similar in structure to the calcium-bound form of calmodulin^16^ and troponin C^17^ (Supporting Information Fig. 4). The orientation of the first helix is, however, very different from that normally observed in “open” EF-hand domains possibly, in part, because of the truncation of the loop between the first two helices. In the PC-2 domain the first helix is orthogonal to the equivalent helix in regular open EF hand domain structures and makes extensive contacts to the C-terminal helix. As such, it can be seen that the N-terminal helix appears to fill the hydrophobic patch used to typically bind other proteins or cognate partners.^17^

## Materials and Methods

### Domain architecture of polycystin-2

The domain architecture of the C-terminus of PC-2 was analyzed using the programs JPRED^18^ and Phyre^19^ to identify regions likely to have a discrete fold. Sequence alignments were generated using CLUSTALW.

### Expression and purification of PC-2 EF-hand domain

The C-terminus of PC-2 and mutant protein domains were cloned into a modified pRSETa^20^ vector containing a thrombin cleavage site to remove the poly-histidine tag and expressed in C41 cells grown in 2xTY medium. Isotopically-labeled protein was prepared by growing cells in K-MOPS minimal media^21^ containing ^15^NH_4_Cl and/or [^13^C]-glucose. Protein was purified using Ni-NTA superflow resin and affinity chromatography. Following thrombin cleavage and further Ni-NTA affinity chromatography unbound protein was applied to a Superdex 75 gel-filtration column. Double de-ionised water was used to make the buffer solutions. The buffer solutions were then passed through Chelex® 100 resin, analytical grade (Bio-Rad Laboratories, Hemel Hempstead HP2 7DX, UK). This was to remove any residual calcium in the buffer solutions. EDTA treated and thoroughly washed plastic containers were always used.

### Isothermal titration calorimetry

All ITC measurements were carried out at 25°C on a VP-ITC system (MicroCal). All solutions were prepared with Milli-Q® water and degassed before 1 m*M* CaC1_2_ solution (in the syringe) was titrated into 10–20 µM protein in the sample cell (1.44 mL), using 4 or 10 µL of titrant per injection over 12 or 30 s. The protein and calcium solutions (1 m*M*) were in 20 m*M* HEPES buffer at pH 7.5. Time intervals of 420–600 s were used between injections to allow complete thermal equilibration. The data were fitted to a one site model using a nonlinear least-squares algorithm packaged with the Origin® software, provided with the VP-ITC system, The stoichiometry (*N*), association constant *(K*_a_*),* and enthalpy change *(ΔH*) were each obtained directly from the titration data. The dissociation constant *(K*_d_*)* was calculated (Supporting Information Fig. S1).

### NMR spectroscopy

Protein samples prepared for NMR spectroscopy experiments were typically 0.5 m*M* in 90% H_2_O, 10% D_2_O, containing 20 m*M* Tris, pH 7.0, 100 m*M* NaCl, and 5 m*M b*-mercaptoethanol. All spectra were acquired using either a Bruker DRX800 or DRX600 spectrometers equipped with pulsed field gradient triple resonance at 25°C, and referenced relative to external sodium 2,2-dimethyl-2-silapentane-5-sulfonate (DSS) for proton and carbon signals, or liquid ammonia for that of nitrogen. Assignments were obtained using standard NMR methods using ^13^C/^15^N-labeled, ^15^N-labeled, 10%^13^C-labeled and unlabeled PC-2 EF-hand NMR samples.^22^,^23^ Backbone assignments were obtained using the following standard set of 2D and 3D heteronuclear spectra: 1H-15N HSQC, HNCACB, CBCA(CO)NH, and ^1^H-^13^C HSQC. Additional assignments were made using 2D TOCSY and DQF-COSY spectra. A set of distance constraints were derived from 2D NOESY spectra recorded from a 1.0 m*M* sample with a mixing time of 100 ms. Hydrogen bond constraints were included for a number of backbone amide protons whose signals were still detected after 10 min in a 2D ^1^H-^15^N-HSQC spectrum recorded in D_2_O at 278 K (pH 5.0). Candidates for the acceptors were identified using the program HBPLUS for the hydrogen bond donors that were identified by the H–D exchange experiments. When two or more candidates of acceptors were found for the same donor in different structures, the most frequently occurring candidate was selected. For hydrogen bond partners, two distance constraints were used where the distance ^(D)^H-O^(A)^ corresponded to 1.5–2.5 Å and ^(D)^N-O^(A)^ to 2.5–3.5 Å. Torsional angle constraints were obtained from an analysis of C′, N, C_α_ H_α_ and C_β_ chemical shifts using the program TALOS.^24^ The stereospecific assignments of H_β_ resonances determined from DQF-COSY and HNHB spectra were confirmed by analyzing the initial ensemble of structures. Stereospecific assignments of H_γ_ and H_δ_ resonances of Val and Leu residues, respectively, were assigned using a fractionally ^13^C–labeled protein sample.^25^ Residual dipolar couplings were measured for proteins aligned in 5% C12E5/1-hexanol. Initial modeling was performed using CS-Rosetta and included the measured RDC values. The three-dimensional structures of the PC-2 EF-hand domain were calculated using the standard torsion angle dynamics-simulated annealing protocol in the program CNS 1.2.^26^ Alignment tensor values for the RDC constraints were calculated using SSIA and the RDC constraints were incorporated in the final round of structure calculations. Structures were accepted where no distance violation was greater than 0.25 Å and no dihedral angle violations >5°. The final coordinates have been deposited in the Protein Data Bank (PDB accession no. 2Y4Q).

## Conclusions

In this study, we have determined a high-resolution structure of the human polycystein-2 EF-hand domain. Based on this, it would appear that this module is derived from a protein originally containing two EF motifs that has lost the first calcium-binding site. Loss of this site prevents the PC-2 domain from binding calcium cooperatively, a common feature of many EF hand proteins. The PC-2 domain also differs from the majority of EF hand containing proteins in its affinity for calcium apparently as a result of a conserved substitution in the EF hand loop. Recently, it has become clear that primary cilia are specialized calcium signaling organelles with a calcium concentration distinct from that of the cytosol. The polycystin channels role appears to be to control the calcium concentration within in primary cilia and thereby regulate signaling pathways linked to this organelle. It is likely that the atypical calcium binding properties of the EF hand domain from PC-2 may have evolved to allow the sensing of a difference range of calcium concentrations in the cilium, to that found in the cytoplasm.^27^

Loss of the first calcium-binding site in the PC-2 domain has been accompanied by a rearrangement of the N-terminal helix that allows it to make more extensive contacts with the rest of the structure. This has important functional implications as the first helix now blocks the hydrophobic binding site used by other open conformation EF hands to interact with ligands. At present, it is unclear how the PC-2 EF hand domain transduces changes in calcium concentration. As, in this protein, calcium binding is coupled to folding many features of the folded conformation could in principle mediate the response to changes in calcium concentration, or potentially simple compaction of the chain due to the folding processes could juxtapose other elements of the protein or their binding partner closer to the channel. It has been shown that other regions of the cytoplasmic tail of PC-2 also bind calcium and the changes induce in PC-2 in response to changes in calcium concentration are probably complex. The structure reported here can provide a basis for detailed structure function studies of the role of this region in the regulation of polycystin that will lead to a clear understanding of the molecular basis of PKD.
