# Supplementary figures and images for: A high-resolution structure of the EF-hand domain of human polycystin-2
Source: Protein Sci. 2014 Jul 2;23(9):1301–8. doi: 10.1002/pro.2513 (PMC4244000; doi:10.1002/pro.2513)

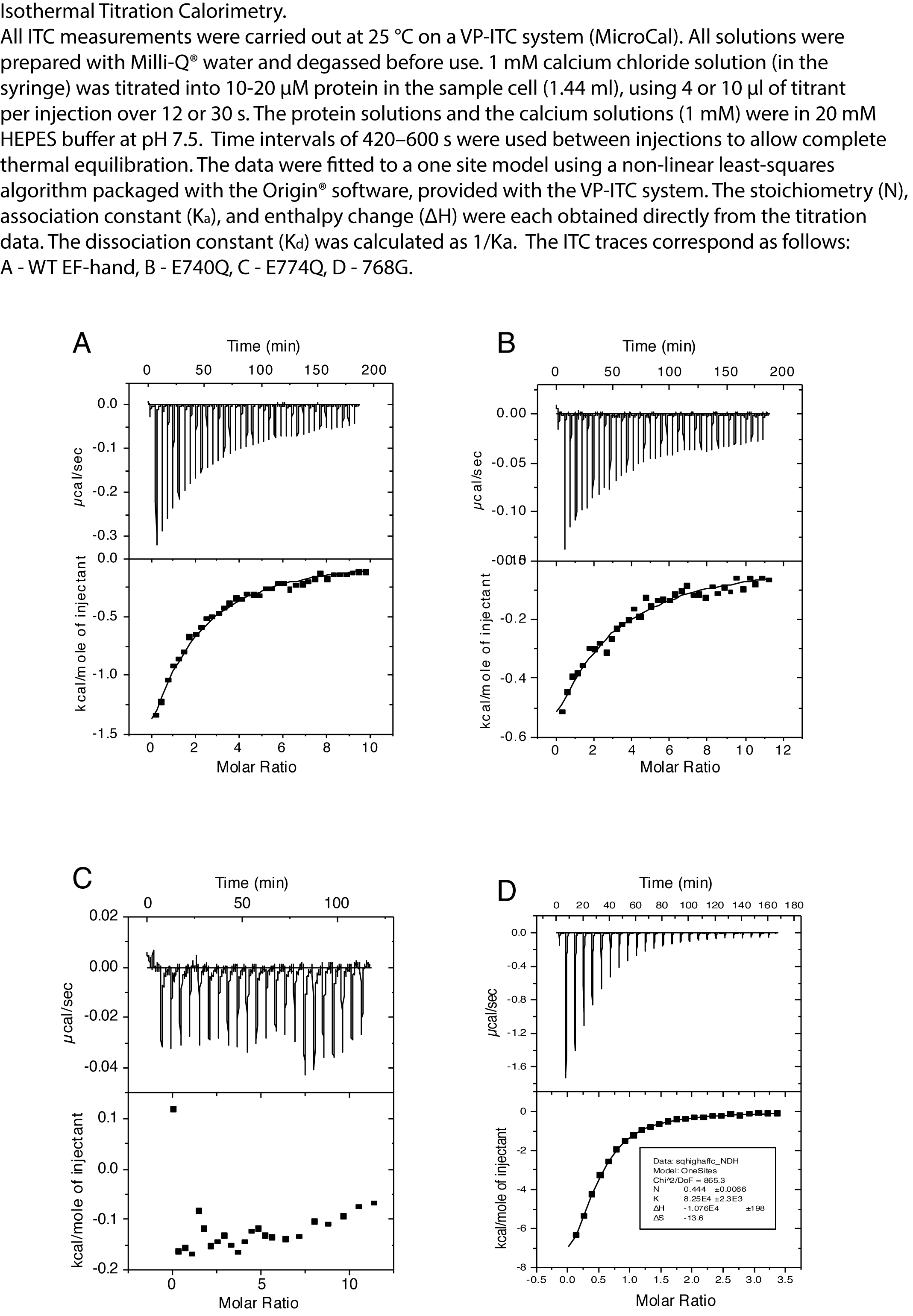

Supplement: Supplementary file 1 — Supplementary Information [file pro0023-1301-sd1.tif]

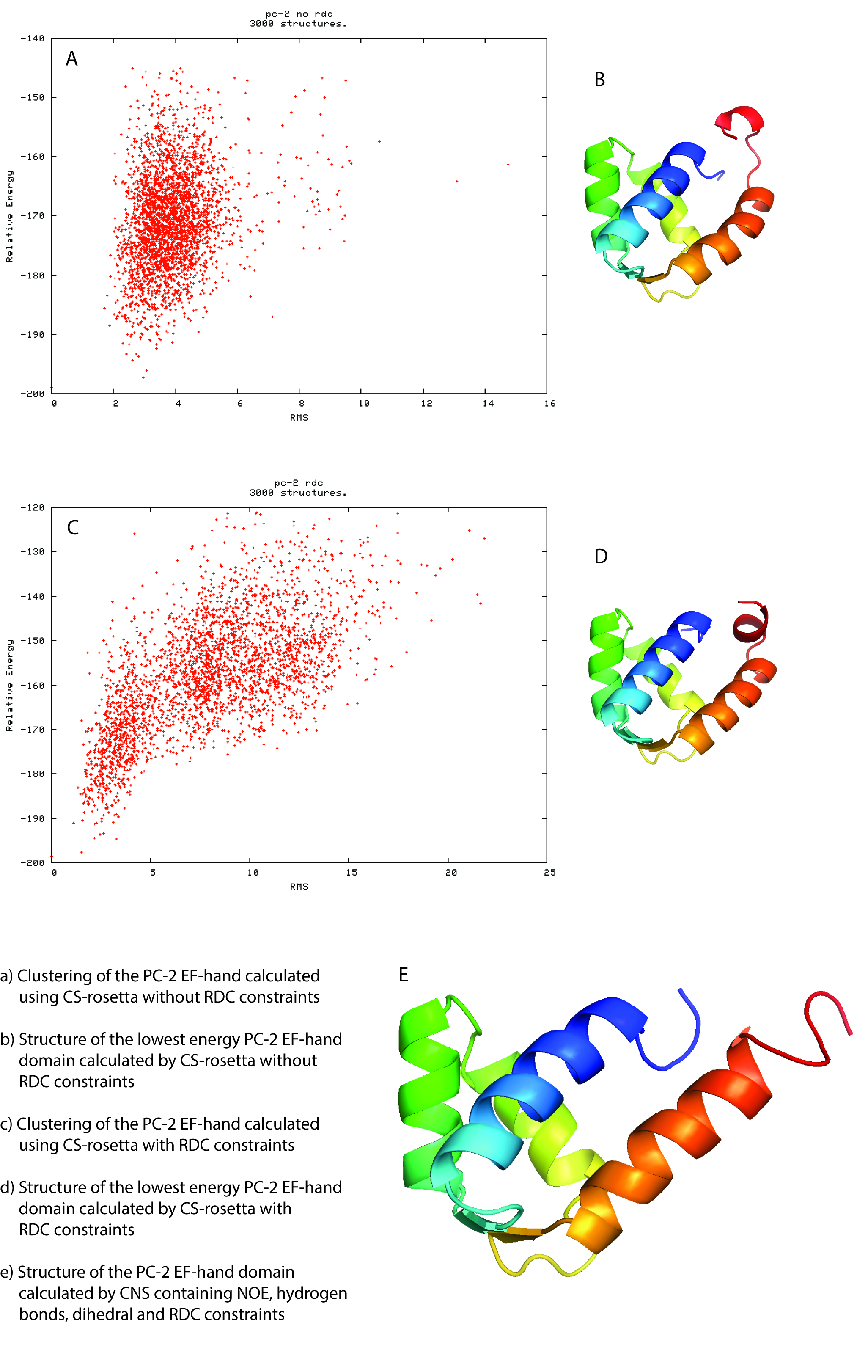

Supplement: Supplementary file 2 — Supplementary Information [file pro0023-1301-sd2.tif]

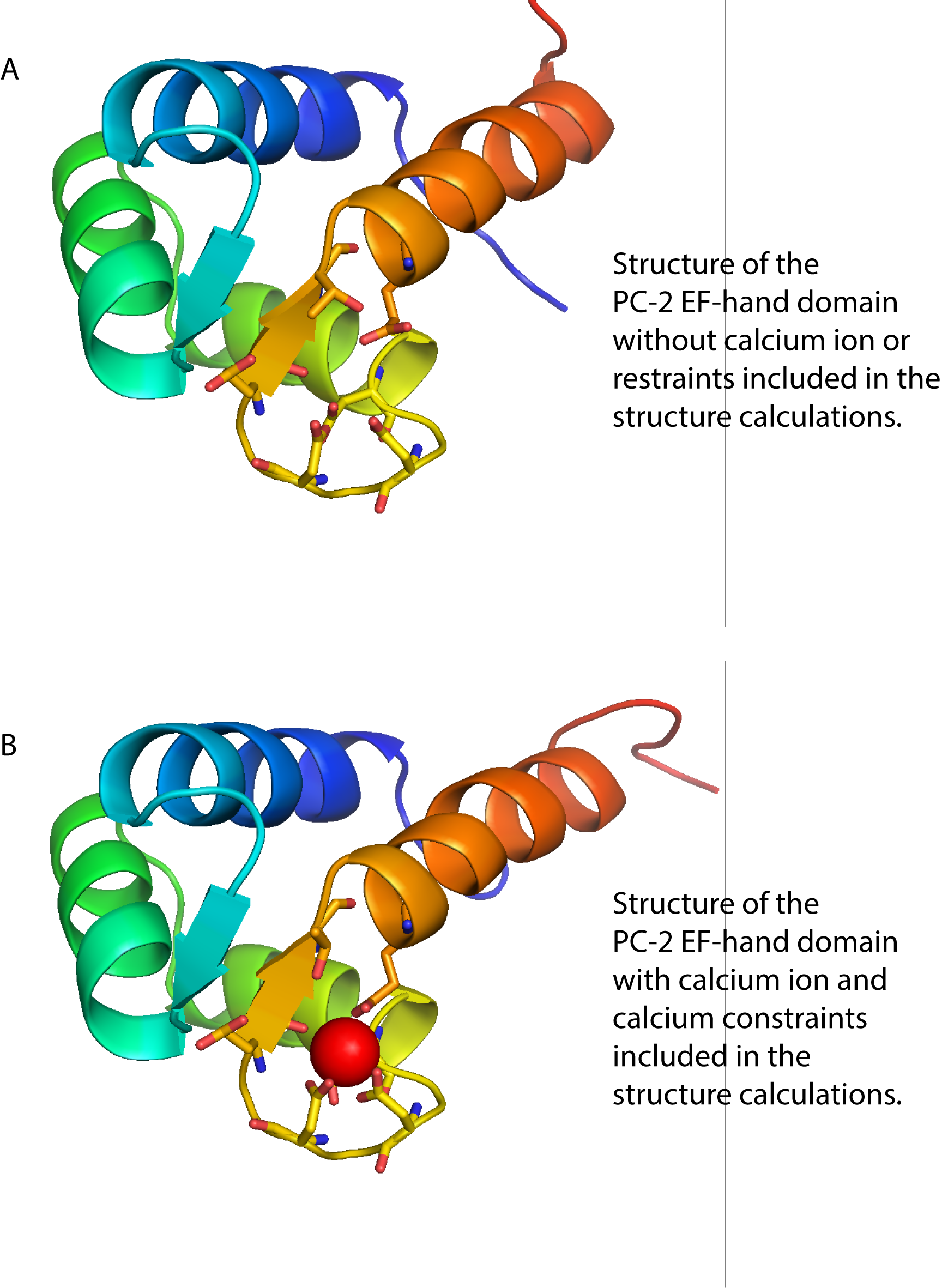

Supplement: Supplementary file 3 — Supplementary Information [file pro0023-1301-sd3.tif]

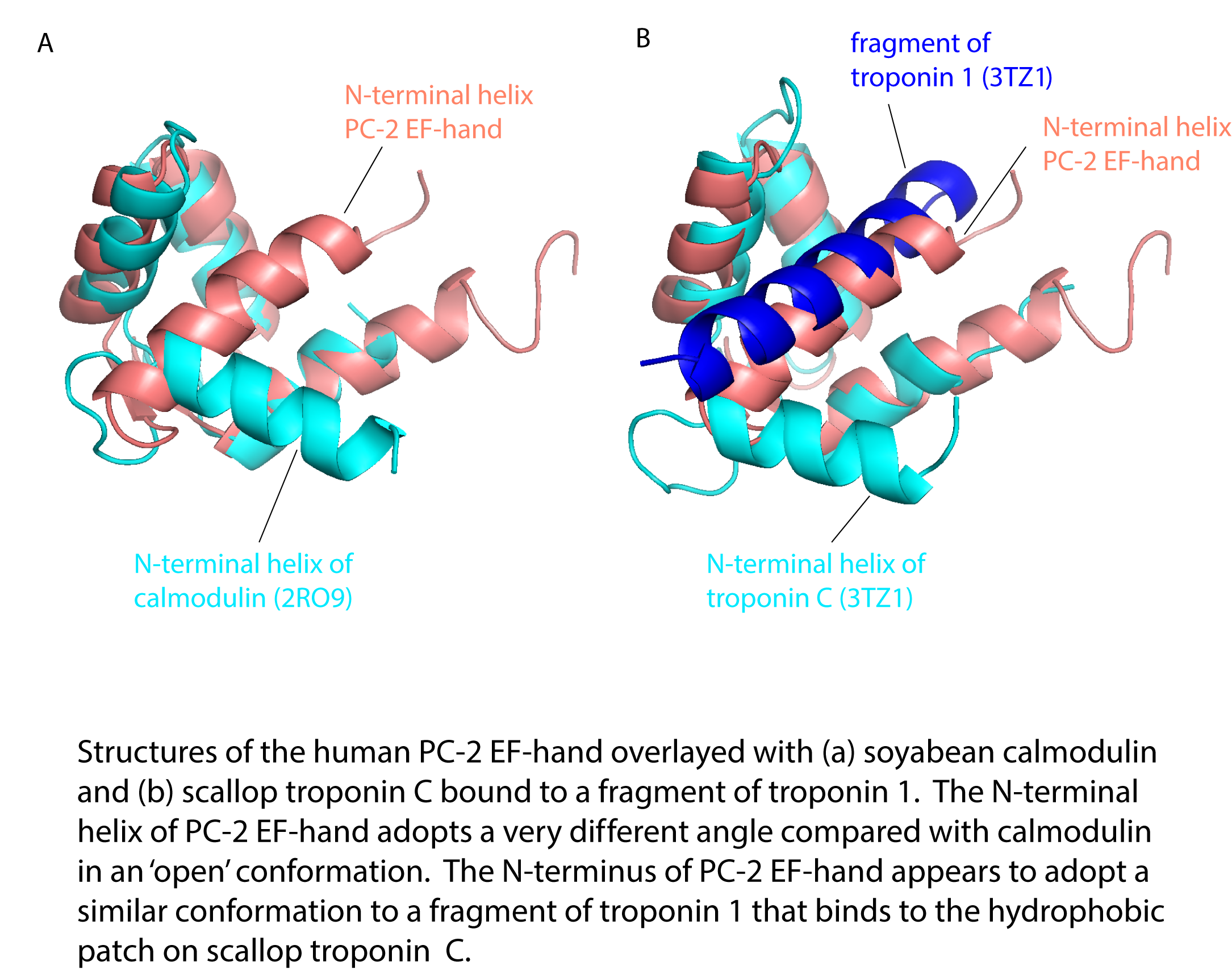

Supplement: Supplementary file 4 — Supplementary Information [file pro0023-1301-sd4.tif]
